# Assessment of angle velocity in girls with adolescent idiopathic scoliosis

**DOI:** 10.1186/1748-7161-4-20

**Published:** 2009-09-16

**Authors:** Ferran Escalada, Ester Marco, Esther Duarte, Josep Ma Muniesa, Roser Boza, Marta Tejero, Enric Cáceres

**Affiliations:** 1 Physical Medicine and Rehabilitation, Hospital de l'Esperança, Institut Municipal d'Assistència Sanitària, Barcelona, Spain; 2 Orthopaedic Surgery Department, Hospital de l'Esperança, Institut Municipal d'Assistència Sanitària, Barcelona, Spain

## Abstract

**Background:**

Although it has been demonstrated that the peak height velocity (PHV) is a predictive factor of progression in adolescent idiopathic scoliosis (AIS), little is known about the usefulness of angle progression in clinical practice. The purpose of this study was to establish a relationship between height and angle velocities, as well as to determine if peak angle velocity (PAV) occurs at the same time than PHV.

**Methods:**

A retrospective study of a cohort of girls with idiopathic scoliotic curves greater than 10°. Data of 132 girls who participated in a previous retrospective study about growth in AIS were used to calculate height and angle velocities. Relationship between height and angle velocities was estimated by the use of a Linear Mixed Model.

**Results:**

PHV and PAV take place simultaneously 1 year before menarche in progressive curves managed with a brace in AIS. Changes in angle velocity are influenced by changes in height growth velocity, in such a way that as from 6 months post-menarche, height growth velocity in this group of girls estimates curve progression velocity (β-coefficient -0.88, p = 0.04).

**Conclusion:**

As from 6 months post-menarche, there is an inverse relationship between height velocity and curve progression in the group of AIS girls with progressive curves managed with a brace. Because height velocity is decreasing from 1 year before menarche, this finding corroborates that at the end of puberty, there is still a risk of progression in this group of girls despite bracing. The assessment of both height and angle velocity might be useful in clinical practice at the time of assessing brace effectiveness and how long bracing has to be indicated.

## Background

Many indicators of skeletal maturity and spinal growth have been proposed, such as chronological age, skeletal age of hand and wrist, Risser's sign, timing of menarche, peak height velocity (PHV) and others [1-8]. None of those has been proved to be accurate enough to predict spinal growth potential in adolescent idiopathic scoliosis (AIS). The PHV, defined as the most rapid growth rate during the early adolescent period, has been shown to have an important predictive value in curve progression much better than other maturity scales such as chronological age, age of menarche or Risser's sign [7]. Timing of the PHV provides valuable information on the likelihood of curve progression, but requirements for measuring the peak are difficult to achieve in clinical practice. Therefore, the search of new indicators is not over [9,10] and their knowledge might contribute to develop new methods to evaluate the end of the spinal growth.

The relationship between growth and curve progression still remains a controversial question in idiopathic scoliosis research. Although it is widely accepted that curve progression is driven to a great extent by spinal growth [1,11], we are not aware of any evidence reporting that angle growth occurs at the same time as height growth. Working on the hypothesis that there is a relationship between height growth and curve progression, it would be reasonable to expect to also find a correlation between height velocity and angular velocity in such a way that when height growth velocity increases, so does curve progression velocity. Arising questions such as if the timing of peak angle velocity could be predictive of curve progression or if the use of PAV could be an indication of success or failure of brace management can not be answered at present.

The purpose of this study was to demonstrate a statistical relationship between height growth and curve progression as well as to determine the time of occurrence of the peak of maximum angle progression in AIS.

## Methods

Data on cohort of 132 girls controlled in a rehabilitation clinics from 1990 to 2001 who participated in a retrospective study about growth in AIS previously carried out in our institution were eligible for inclusion. Although the methodology and procedures are all described in detail in our previous report [12], the most interesting points regarding Material and Methods are summarized as follows.

There were 389 children evaluated during this period of time, from which only 132 met the inclusion criteria:

- Girls with AIS

- Curve magnitude of more than 10° as measured by the Cobb method[13]

- Date of menarche well documented

- Minimum of 4 visits to our institution over a at least 2 years around the time of occurrence of menarche

The time interval subject to study was from 2 years before to 5 years after menarche. Height and Cobb angle measurements were performed at 6 (±3) month intervals. Height measurements were recorded by the nursing stuff. Patients to be measured hat to be standing, looking straight ahead and without wearing shoes or brace. Height was recorded in centimeters using a wall-mounted ruler with a perpendicular slide. The Cobb angle was measured from X-rays by a single senior clinician who carried out two measurements in order to reduce intraobserver variability [14]. All radiographs were posteroanterior projection with the patient standing and were always taken out of the brace in follow-up evaluations.

For the purpose of this study, the main variables were calculated from height and Cobb angle measurements:

- Height growth velocity (height increase divided by the time interval between two consecutive medical controls, expressed in centimetres per year):



- Angular velocity (angle increase divided by the time interval between two consecutive medical controls, expressed in angle degrees per year):



Other variables recorded for each visit were:

- Treatment management categorized into three groups: observation, bracing and/or surgical treatment

- Progression of the curve, progressive curves being defined as those increasing by at least 5 degrees per year during the follow-up time

Treatment management was recorded as a variable with 3 categories: observation, bracing with a thoracolumbosacral orthoses (TLSO) and/or surgical treatment. Patients observed over a period of time who later required bracing were first included in the observation group until the time that the brace was prescribed, and then transferred to the orthosis group as of that time. There were 3 girls initially treated with a brace who dropped out of the study when surgical treatment was required.

Controls were performed at time intervals of 6 (+/- 3) months. For the purpose of this study, 1 year was assumed to be 365 days and expressed as a decimal variable in which 6 months equals to 0.5 years. An example of the calculation of height and angular velocities for an individual patient is shown in Table 1. Comparisons between girls were performed by the time of occurrence of menarche instead of using chronological ages. During the first 2 years, when the date of menarche was obviously not known, controls were also carried out with a minimum regularity of 6 months. If a particular patient required more controls, the rest of measurements were obviated.

**Table 1:** General characteristics of the sample (n = 132).

Age at time of diagnosis	11.6 (SD 2.47) years
Menarche age	12.8 (SD 1.18) years
Curve type:	
Thoracic	25 (18.9%)
Lumbar	51 (38.6%)
Thoracolumbar	25 (18.9%)
Double curves	31 (23.4%)
Side	
Right	68 (51.5%)
Left	64 (48.5%)
	
Curve length (number of vertebrae)	
4 vertebrae	13 (9.8%)
5 vertebrae	47 (35.6%)
6 vertebrae	33 (25.0%)
7 vertebrae	24 (18.1%)
8 vertebrae	9 (6.8%)
9-13 vertebrae	6 (4.5%)
	
Cobb angle (at time of menarche)	20.3 (SD 11.42)
	
Type of treatment:	
Observation	88 (66.7%)
Bracing	44 (33.3%)

### Statistical analysis

Categorical variables are presented as percentage and absolute values. Quantitative variables are presented with their mean and standard deviation. Changes in angle velocity were evaluated in a Linear Mixed Model. The method used was a Restricted Maximum Lilkelihood where angle velocity was the dependent variable. Height growth velocity was included as a predictor, patient number as a random effect and moment as a fixed effect. Statistical analysis was performed by R-software version 2.62 [15]. The level of statistical significance was 0.05 for all hypothesis comparisons.

## Results

The mean follow-up time was 39.6 (SD 17.6) months (range: 18-87 months). The mean age at the time of diagnosis was 11.6 (SD 2.5) years and the mean menarchal age was 12.8 (SD 1.9) years. The most frequently observed curves were lumbar (38.6%) with an average length of 6.0 (SD 1.5) vertebrae. Of the 132 participants in the study, 88 (66.7%) girls were managed with observation and only 44 girls (33.3%) were managed with a brace. The general characteristics of the sample are detailed in Table 1.

As detailed in Table 2, the magnitude of the curve was significantly higher in the braced group. Mean Cobb angle was 11.8 (SD 3.1) degrees (max 20, min 10) for the girls managed with observation and 25.0 (SD 6.21) degrees (minimum and maximum values 14 and 37, respectively). Lumbar curves were more frequent in the observation group whereas double curves were for the braced girls (p < 0.001).

**Table 2:** Distribution of curve patterns and Cobb angles (n = 132)

	**Observation group**	**Braced group**	**p**
	**(n = 88)**	**(n = 44)**	
Curve type:			
Lumbar	41 (46.6%)	10 (22.7%)	
Thoracic	16 (18.2%)	9 (20.5%)	>0.05
Thoracolumbar	17 (19.3%)	9 (20.5%)	>0.05
Double curve	14 (15.9%)	16 (36.4%)	<0.001
			
Cobb angle:			
- At time of maximal angle growth (1 year before menarche)	11.8 (SD 3.1)	25.0 (SD 6.21)	<0.001
- At time of menarche	13.4 (SD 4.46)	24.0 (SD 12.28)	<0.001

**Figure 1:**
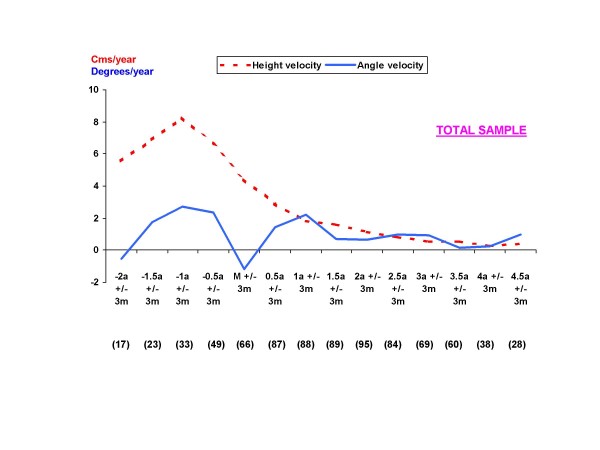
**Graph showing the mean values for height and angle velocities from the current study of 132 girls with idiopathic scoliosis**. The values in parentheses are the number of patients with a height and angle velocity measurement for each period.

In the total sample, PHV occurs at 1 year before the onset of menses (Figure 1). In the graphic assessment of mean angular velocities, a PAV is also observed at 1 year before menarche, followed by a negative deceleration in angle growth around the perimenarchal period.

**Figure 2:**
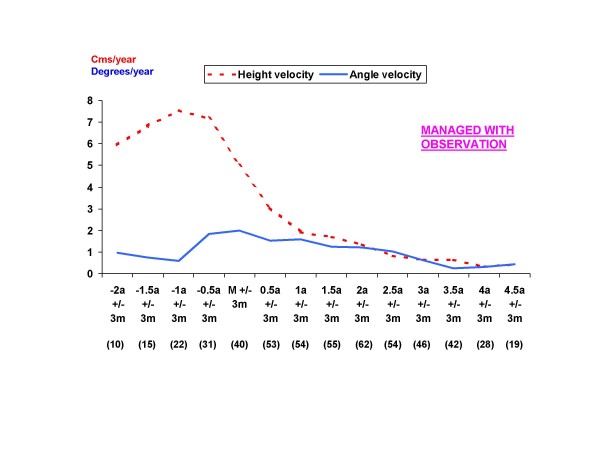
**Graph showing the mean values for height and angle velocities from the current study of girls with idiopathic scoliosis managed with observation**. The values in parentheses are the number of patients with a height and angle velocity measurement for each period.

**Figure 3:**
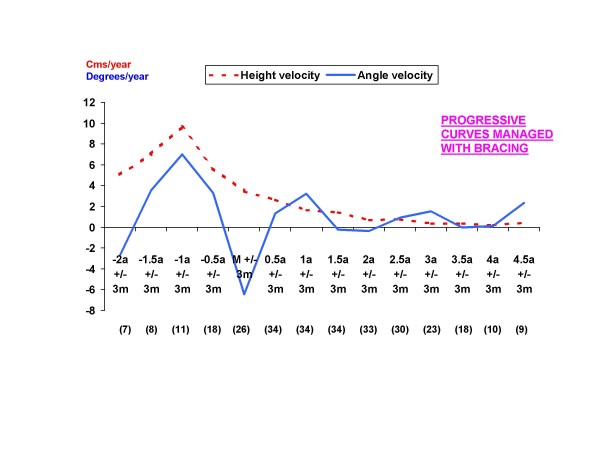
**Graph showing the mean values for height and angle velocities from the current study of girls with idiopathic scoliosis managed with bracing**. The values in parentheses are the number of patients with a height and angle velocity measurement for each period.

The same analysis was performed by dividing the sample into two groups according to treatment type: curves managed with observation and progressive curves managed with a brace. Height velocity has the same behaviour as the one observed for the general sample with a PHV occurring at 1 year before menarche in both groups. As for angle velocity, no peak velocity was obviously observed in the observation group (Figure 2), whereas for girls with AIS who did require bracing, the PAV occurs at the same time as PHV, followed by a deceleration of angle growth in the perimenarchal period (Figure 3).

The search of correlation coefficients between height velocity and angle velocity is a very complicated matter when studying children with scoliosis. This is due overall to the different progression patterns: height grows following a linear pattern while angle growth is in spurts. Because a significant interaction (p = 0.01) was observed between the use of brace and the effect of height velocity on the angle velocity, data was split in users and no users of a brace. The β-coefficient values resulting from the Restricted Maximum Likelihood are detailed in Table 3. In the premenarche period (from 2 years to menarche), no relationship was found between height and angle velocities in observed and braced girls. Nevertheless, in the second period considered (menarche to end of follow up), every increase of a height velocity unit leads to a decrease of 0.88 angle velocity units indicating that there is an inverse relation between height and angle velocities in the group of girls managed with a brace (β-coefficient -0.88, p = 0.04).

**Table 3:** Relationship between angle and height velocities

	**Braced group**		**Observation group**	
	**β-coef**	**p**	**β-coef**	**p**
Premenarche period (2 years to menarche)	-0.17	0.704	0.14	0.345
Post-menarche period (menarche to end of follow up)	-0.88	0.043	0.12	0.193

## Discussion

This study examines the time of occurrence of peak angular velocity in a group of girls with AIS and looks for correlations between height velocity and angle velocity.

Before attempting any further discussion of these findings, some limitations of the present study should be noted. Many shortcomings appear whenever scoliosis and/or growth are studied [14,16-20] and they are probably the reason for the shortage of prospective and conclusive studies on this subject. Prospective works are difficult to perform because of the long follow-up period and this is why most papers in medical literature are transversal studies or retrospective cohort studies. The work by Nachemson *et al *[14,20] deserves especial mention as the only prospective work on the effectiveness of bracing in the treatment of AIS.

It is very difficult to form homogeneous samples as growth in girls with the same chronological age is very variable. Little [7] demonstrated that the height pattern is similar among girls with AIS when they are grouped according to PHV. Considering that in many cases, this PHV is unknown, for the purpose of this study girls were grouped using the date of menarche as the reference for comparing patients. Although mean age of diagnosis for this sample was 11.6 years (approximately 1 year before occurrence of menarche (mean 12.8, DE 1.2)), note that diagnosis was made in first evaluation 1 or 2 years after menarche in 50% of girls. Our results, as well as the data presented by Little [7] show that menarche occurs one year after PHV. Therefore, considering that the mean age of diagnosis was 11.6 years, this means that at the beginning of follow-up, the PHV had already taken place in 50% of cases.

Contrary to what the first observations [21] seemed to indicate, curve progression in idiopathic scoliosis is not linear. It is frequent to observe curves that remain stable for a time and from a point, and then start to progress. On the other hand, it is not unusual to observe short periods of progression in a growth spurt followed by stabilization. When studying angular velocities, a repeated angle increase of 0 was frequently observed, even in girls with progressive curves. This represented a handicap for the use of both parametric and non-parametric statistical tests and specially, those for univariate analysis. This conditioned the fact that, when searching for a correlation, calculations of r and R^2 ^were not feasible and the authors had to make an approximation by using absolute values of height and angle instead of the height and angle velocities [10].

The authors would like to mention the inference of the brace in this sample. Although the period after menarche is a phase of growth deceleration, the effect of the brace is, in our opinion, partially responsible for the deceleration velocity after menarche in braced curves. As observed in Figures 1 and 3, there is an important decrease in angular velocities in the perimenarchal period followed by a second growth spurt which occurs during the 6-12 months after menarche. Even though a period called *âge heureux *(happy period) consisting of a period of a relative stabilization of the curve, followed by a second growth spurt has been described [22], we think that in the sample, this decreasing velocity was due to the brace effect. It is not unusual to observe important initial reductions with the use of the brace, and in posterior controls, to find that the curve has returned to its initial values. If the second peak of PAV observed in graphs correlates to the cessation of bracing in these patient is another question that deserves further research.

In this study, graphic assessment suggests a relationship between height growth velocity and curve progression velocity, though frequent progression in spurts did not allow a statistical relationship to be found between angle and height velocities. Nevertheless, this problem was partially solved by correlating absolute values of height and angle over a time interval. In our previous work [10], a statistically significant correlation between growth rates was noted up until 2.5 years after menarche, specially in AIS managed by observation (r = 0.632, p < 0.001).

## Conclusion

In summary, we conclude that both PHV and PAV occur at the same time (1 year before menarche). Over this time is when most of bracing treatments are prescribed and when the brace is expected to have a maximum effect on angle velocity. Therefore, at 6 months post-menarche, there is an inverse relationship between height and angle velocities in the group of AIS girls with progressive curves managed with a brace. Considering this is a period of height deceleration, this finding indicates there is still a risk of progression in girls with moderate-severe curves despite bracing. The assessment of both height and angle velocity might be useful in clinical practice at the time of assessing brace effectiveness and how long bracing has to be indicated.

## Competing interests

The authors declare that they have no competing interests.

## Authors' contributions

FE conceived and designed the study, performed analysis and interpretation of data, carried the assessments and gave final approval of the version to be published. EM contributed to acquisition, analysis and interpretation of data and was involved in drafting the manuscript. ED and JMM revised critically for important intellectual contents. RB and MT contributed to acquisition of data and analysis EC participated in its design, revised critically for intellectual contents and gave final approval of the version to be published. All authors read and approved the final manuscript.
