# Correction: Assessment of angle velocity in girls with adolescent idiopathic scoliosis

**DOI:** 10.1186/1748-7161-4-23

**Published:** 2009-10-10

**Authors:** Ferran Escalada, Ester Marco, Roser Belmonte, Esther Duarte, Josep Ma Muniesa, Roser Boza, Marta Tejero, Enric Cáceres

**Affiliations:** 1 Physical Medicine and Rehabilitation, Hospital de l'Esperança, Institut Municipal d'Assistència Sanitària, Barcelona, Spain; 2 Orthopaedic Surgery Department, Hospital de l'Esperança, Institut Municipal d'Assistència Sanitària, Barcelona, Spain

## Abstract

Correction to Escalada F, Marco E, Duarte E, Muniesa JM, Boza R, Tejero M, Cáceres E. Assessment of angle velocity in girls with adolescent idiopathic scoliosis. Scoliosis 2009; 4:20.

## Correction

After publication of this work [1], we noted that we inadvertently failed to include the complete list of all coauthors. The full list of authors has now been added and the Authors' contributions and Competing interests section modified accordingly.

## Competing interests

The authors declare that they have no competing interests.

## Authors' contributions

FE conceived and designed the study, performed analysis and interpretation of data, carried the assessments and gave final approval of the version to be published. EM contributed to acquisition, analysis and interpretation of data and was involved in drafting the manuscript. RBe contributed to design, analysis and interpretation of data and reviewed the article critically for important intellectual content. ED and JMM revised critically for important intellectual contents. RBo and MT contributed to acquisition of data and analysis. EC participated in its design, revised critically for intellectual contents and gave final approval of the version to be published. All authors read and approved the final manuscript.
